# Comparative analysis of bone consolidation chronology in claw toes operated through minimal invasive osteotomies in diabetic vs. non-diabetic patients

**DOI:** 10.3389/fsurg.2022.1027094

**Published:** 2022-12-12

**Authors:** Cristina Batalla-Salgado, Javier Ferrer-Torregrosa, Francisco Muñoz-Piqueras, Miguel Muñoz Bautista, Carlos Barrios

**Affiliations:** ^1^ Doctorate School, Valencia Catholic University “San Vicente Mártir”, Valencia, Spain; ^2^ Podiatry Department, School of Medicine and Health Sciences, Valencia Catholic University “San Vicente Mártir”, Valencia, Spain; ^3^ Surgery Unit of Piqueras Clinic, Madrid, Spain; ^4^ Institute for Research on Musculoskeletal Disorders, School of Medicine and Health Sciences, Valencia Catholic University “San Vicente Mártir”, Valencia, Spain

**Keywords:** claw toe, minimally invasive surgery, diabetic patients, bone consolidation, AOFAS scale

## Abstract

**Objective:**

To compare bone healing time in osteotomies performed in claw toes correction through minimal invasive surgery in diabetic vs. non-diabetic patients. The relation between the patient's ages and the American Orthopedic Foot and Ankle Surgery Society (AOFAS) functional scores before and after surgery was also analyze in the two types of patients.

**Method:**

A series of 45 women, 23 of them suffering from Diabetes Mellitus, were operated to correct claw toes. The surgery was always performed through minimal invasive digital osteotomies. After the intervention, bone healing was controlled by a fluoroscopic weekly follow-up until a complete bone consolidation was reached. Bone healing time was compared in in two groups of patients, diabetic and non-diabetic. All patients were evaluated with AOFAS scale 48 h before and 90 days after the intervention.

**Results:**

The time of bone healing ranged from 24 to 40 days after the surgery and took shorter time of consolidation in non-diabetic patients although the Mann Withney *U* test did not show statistically significant differences (*p* = 0,409, effect size (ES) = 0,14 [-0.20 to 0.45]) between both groups. A statistically significant association (*r* = 0.71, *R*^2 ^= 50%, *p* < 0.001) was found between the healing days and the day of medical discharge, also between the ages of the patients and the medial discharge (*r* = 0.36, *R*^2 ^= 13%, *p* < 0.001). However, no statistically significant associations were found between pre-intervention glycemia and days of bone consolidation, neither in medical discharge (*r* = 0.07, *p* = 0.646 y *r* = 0.07, *p* = 0.648, respectively). AOFAS test scores and the diabetes status showed statistically significant differences, both in the main effect of Diabetes (F_[1,41] _= 9.41, *p* = 0.004) as in the interaction between diabetes and age (F_[1,41] _= 9.17, *p* = 0.004).

**Conclusions:**

The bone healing time in claw toes operated through minimal invasive osteotomy surgery is not influenced by the presence of diabetes. The consolidation speed and the improvement in AOFAS functional scale score post-surgery in diabetic and elder patients was related to duration of the medical discharge.

## Introduction

The prevalence rate of diabetes mellitus (DM) is growing every day according to World Health Organization (WHO) reports (1) and the International Diabetes Federation (2). Since the end of the last century, the number of people with DM has doubled, reaching more than 463 million people last year, with type 2 diabetic patients representing 90% of those with the disease. This fact could reflect a significant lack of control of healthy lifestyle habits such as diet and/or smoking habits (3). On the other hand, epidemiological evidence has shown that the prevalence of this disease will increase up to 700 million people by 2045 (4), as a result, hospital burden resulting from DM treatments can rise steeply.

Particularly, the diabetic foot is one of the most serious DM conditions, with non-traumatic amputation of the limb being one of the major complications (5). A recent meta-analysis conducted by Sen et al. (6), with more than 1,800 patients, showed that male sex, smoking, history of amputation and history of osteomyelitis were the variables most associated with amputation in patients with diabetic foot infections. Therefore, it is important to analyze how to prevent severe complications with both resolutive and prophylactic treatments. In this sense, arthropathies linked to vasculopathy in the diabetic foot are triggers of potential amputations, so that the correction of the former benefits its preservation (7, 8). Among these arthropathies affecting the forefoot, digital deformities (i.e., claw, hammer, and mallet toe) and deformities of the first radius (i.e., hallux valgus) are the most common (9). An investigation leaded by Ababneh et al. (10) with 1,000 patients showed that foot deformities in patients showed that more than 17% had hallux valgus whilst 16% had claw or hammer toes.

Previous studies have shown that osteotomies can help as a treatment to relieve plantar pressures generated by anatomical deformities (11–14), flexor tenotomies for digital alterations (15), as well as gastrocnemius lengthening to unload excessive metatarsal and digital area load (16).

Other studies have described deleterious effects of diabetic hyperglycemia on bone tissue quality (17), for example fragility or energy absorption (18), changes in trabecular volume and thickness (19) as well as mechanical properties (20) in addition to variants in the properties of collagen, minerals, and AGEs (advanced glycation products) (20, 21). The accumulation of these diabetic exposure changes in bone cell activity could impair consolidation and long-term progenitor cell potential and may create vascular deficiencies at the fracture site (22). These effects could be comparable to those caused by aging on bone structural integrity (23). The sum of histological changes that the bone undergoes suggests standardized histomorphometry evaluations by means of radiographic images that allow the assessment of its evolution (24) and the relationship between different groups of patients.

It is important to keep in mind the functional assessment of patients after surgery. In this sense, the American Orthopedic Foot, and Ankle Surgery Society (AOFAS) questionnaire is usually the most used to measure the functional assessment of patients. The score ranges from 0 to 100 depending on the degree of limitation of the patient. A score close to 0 is evidence of poor function, while scores close to 100 show improved foot and ankle function (24, 25).

Given the importance of ulcer prevention in patients with DM and the high incidence of non-traumatic amputations of the foot (26), rapid action by means of an osteotomy to correct claw toes could help to avoid the outcome of amputation (27). However, scientific studies comparing variables such as consolidation time in DM patients are scarce. Therefore, this study aimed to compare the bone consolidation time of osteotomies performed for claw toe correction by minimally invasive surgery in diabetic patients compared to non-diabetic patients. On the other hand, the second objective of the present investigation was to analyze the relationship between patient age and functional scores (i.e., AOFAS) after surgery regarding the patient condition (i.e., diabetic vs. non-diabetic). We hypothesize that the time to consolidation in patients with diabetes (previous normal glucose levels) will be similar compared to patients without diabetes. On the other hand, older diabetic patients will have greater perceived improvement in functionality scores.

## Material and methods

### Participants

Consecutive subjects with a diagnosis of claw toes presenting to a Surgery Unit of Piqueras Clinic, Madrid, Spain. from January 2021 to January 2022 were screened for inclusion in this study. Finally, a total of 49 participants were recruited for this descriptive-correlational study. The mean and standard deviation (SD) of age was 70 ± 9.28 years. From the total sample size, 51.15% (*n* = 23) were diabetic (see Table 1). The eligibility criteria of the present study were, inclusion criteria: a) patient with central claw toes, b) > 50 years and c) women. On the other hand, the exclusion criteria were a) previous foot surgeries (i.e., hallux valgus surgery, hallux rigidus, metatarsal osteotomies, digital osteotomies, exostectomies, tenotomies, capsulotomies), and b) uncontrolled blood sugar levels (i.e., fasting plasma glucose <80 to 130 < mg/dl). All participants were informed about the risks and benefits of participating in the investigation. Subsequently, they signed an informed consent. The study was authorized by the Research Ethics Committee of the Universidad Católica de Valencia San Vicente Mártir (Ref. UCV/2029–2020/159).

**Table 1:** Participants characteristics included in the present study.

Variable	Total	Diabetes		*p*-value
	*n* (SD)	Yes *n* (SD)	No *n* (SD)	
Participants (*n*)	45	23	22	na
Age (years)	70.6 (9.96)	76.59 (6.35)	64.87 (9.46)	< 0.001**
Foot (*n* = Right/*n* = Left)	34/11	18/4	16/7	0.490
Osteoarticular				
Disease				
Arthritis	16/45	11/22	5/23	0.048*
Arthrosis	22/45	15/22	7/23	0.002**
Osteoporosis	3/45	0/22	3/23	0.080
Cardiovascular	45	13/22	9/23	0.181
Disease				
Pre-intervention	95.24 (11.52)	95.96 (11.93)	94.57 (11.35)	0.955
Glucose				
Days consolidation	34 (3.86)	35 (3.64)	34 (4)	0.158
High Days	52 (4.34)	53 (3.75)	51 (4.71)	0.108

SD, standard deviation; na, not applied.

* *p* < 0.05.

** *p* < 0.001.

### Procedure

Prior to surgery, each patient underwent an initial anamnesis according to age, sex, smoker or non-smoker, pathologic status (i.e., diabetic and/or non-diabetic), glycemic value, osteoarticular diseases and cardiovascular alterations and peripheral vascular evaluation with Doppler and sphygmomanometer. Femoral, Popliteal, Posterior Tibial and Pedial pulses were measured, moreover to the Radial pulse for the calculation of the Ankle-Brachial Index (ABI). No alterations were observed in the pulses or ABI; all participants had normal values of 0.9 and 1, so the risk of peripheral vascular disease in the participants was minimal with perfect diabetic control in these patients.

In addition, an assessment of their metatarsophalangeal status by AOFAS scale (28, 29) and pain intensity with a visual analogue scale (VAS) was done (30). The AOFAS clinical scale system allows the assessment of 3 main dimensions: pain (i.e., mild, moderate, and severe), function (i.e., divided into six subscales) and foot alignment (i.e., good, fair, or poor). Previous research has shown the validity and adequate reliability of the AOFAS questionnaire (31, 32). The AOFAS clinical evaluation system was administered 48 h before surgery and 90 days after surgery.

### Surgical procedure

**Figure 1:**
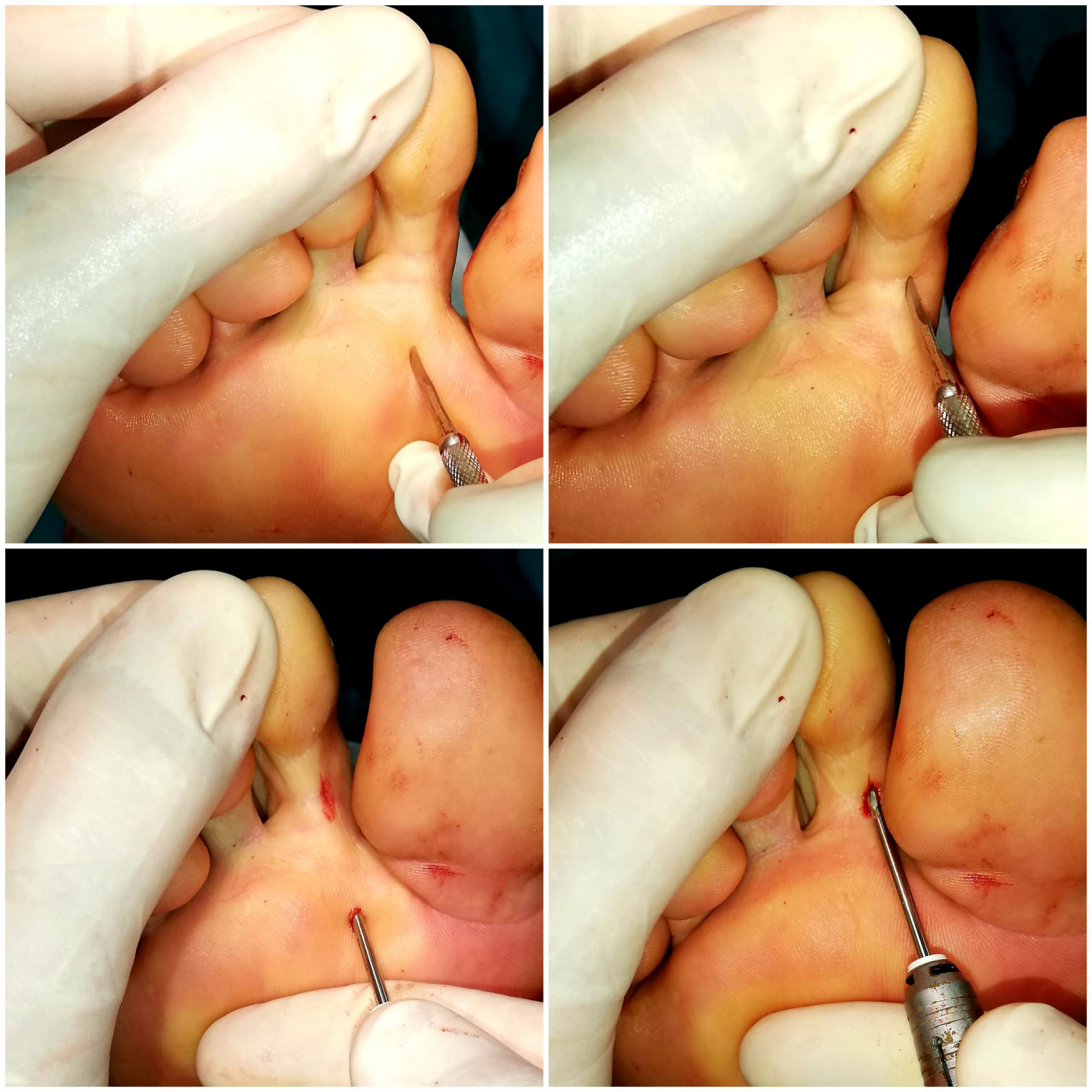
Surgical procedure for the correction of claw toe with osteotomy at the base of the phalanx and claw toe.

**Figure 2:**
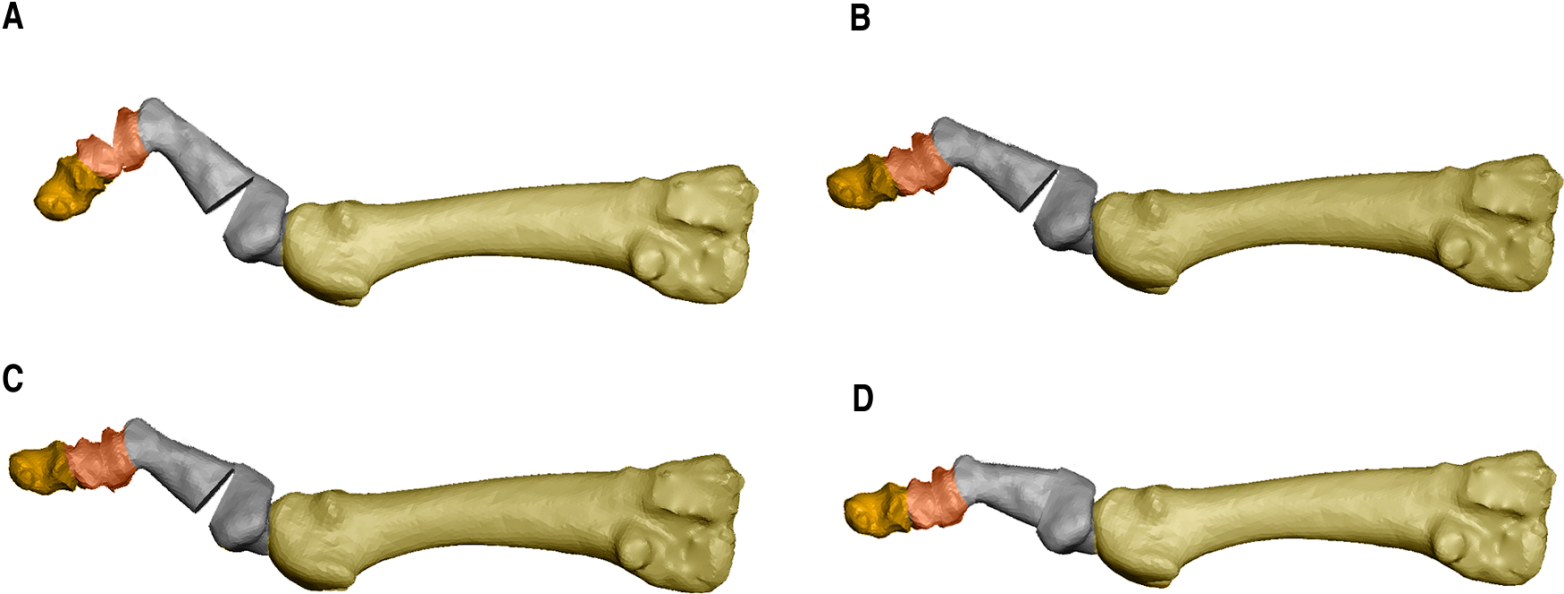
(**A**) design of a double osteotomy of the proximal phalanx at proximal and distal level and an osteotomy of the middle phalanx in case of claw toe; (**B**) closing of the middle phalanx osteotomy; (**C**) closing of the distal osteotomy of the proximal phalanx; (**D**) closing of the proximal osteotomy of the first phalanx with complete reduction of the deformity.

**Figure 3:**
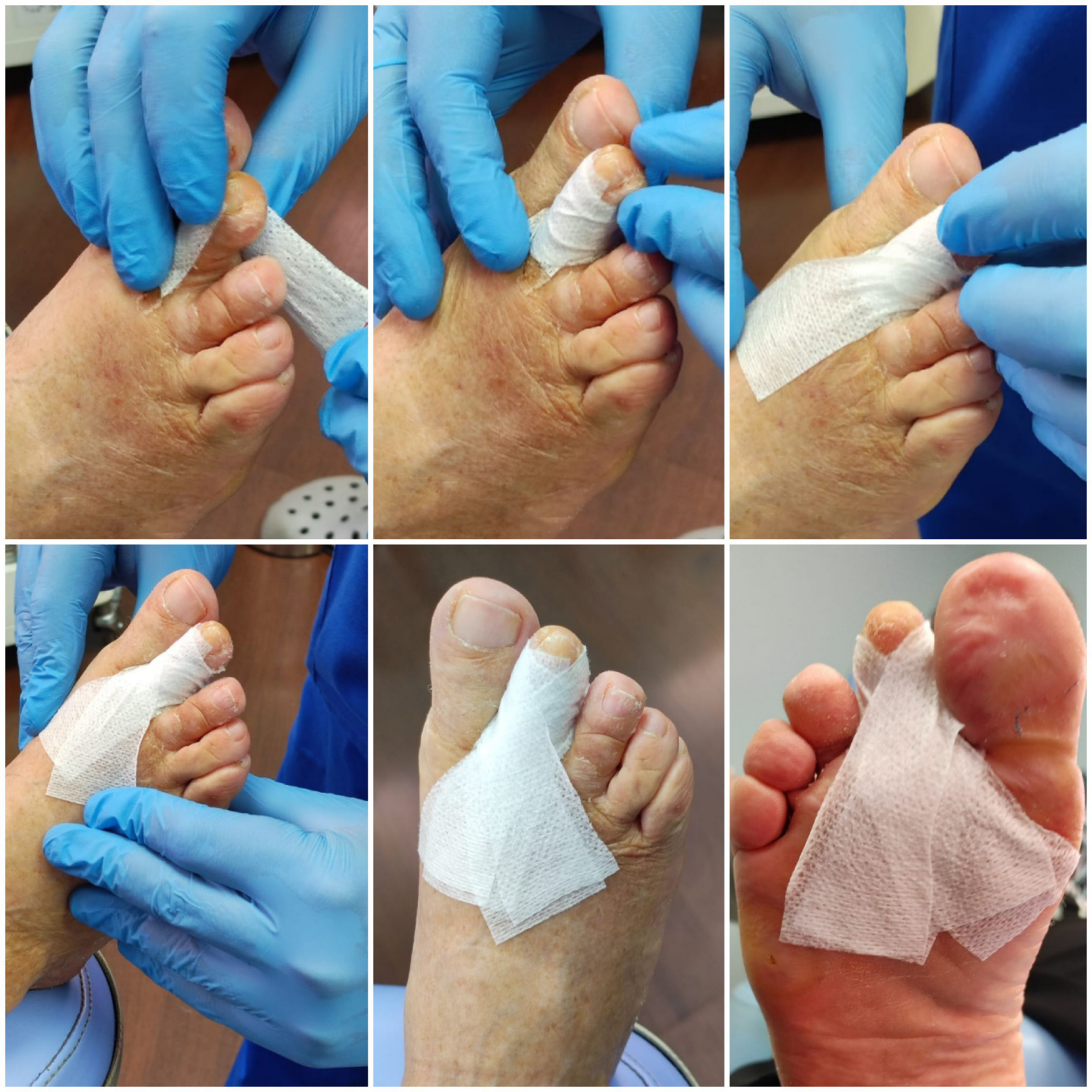
Post-surgical dressing, weekly change.

All patients underwent plantar base osteotomy at the base of the proximal phalanx and dorsal base osteotomy at the middle phalanx, described by Dr. Isham, both by MIS (33–35) (Figure 1 and Figure 2). In some cases, soft tissue release through tenotomies was necessary according to accepted guidelines (35). All procedures were performed under regional anesthesia (Mepivacacia 2% and Bupivacaine 0.5%) of the operated limb by the same surgeon. Finally, a bandage was made to control the osteotomies and the correct alignment of the digits by means of Hypafix® (see Figure 3).

### Post-surgical control

All the surgeries were successfully performed. Each patient who underwent surgery was able to walk immediately after the surgery with the post-surgical shoe (Herbitas ® surgical shoe model, valid for right/left foot) with a flat non-slip sole and Velcro fastening. The first revision by the surgeon took place 48 h after each operation. Each revision had a thorough fluoroscopic control. The patients were supervised on a weekly basis. Bone consolidation was assessed based on the Montoya Scale for post diaphyseal fracture bone callus formation (36, 37), whilst the fluoroscope was used to monitor the evolution of the osteotomies until complete consolidation and recovery of the finger was obtained. The degree of consolidation was classified as grade I mild consolidation, grade II and III moderate consolidation and grade IV total consolidation.

### Statistical analysis

All variables were expressed as a mean and SD. The normality assumption (i.e., Kormogorov-Smirnov test) and homogeneity of variances (i.e., Levene's test) were analyzed. Generally, whenever both assumptions were met, an independent samples *t*-test was performed. If either assumption was not met, the nonparametric version (i.e., Mann-Withney *U*) was performed. The effect size (ES) was calculated using Cohen's formulas, considering an ES < 0.19 trivial, 0.20–0.49 small, 0.50–0.79 moderate and > 0.80 large (18). The relationships between variables were performed using Pearson's or Spearman's correlation coefficient, in case of non-normality, The level of statistical significance was set at *p* < 0.05. All analyses were performed using the statistical analysis software JASP (The Netherlands).

## Results

**Figure 4:**
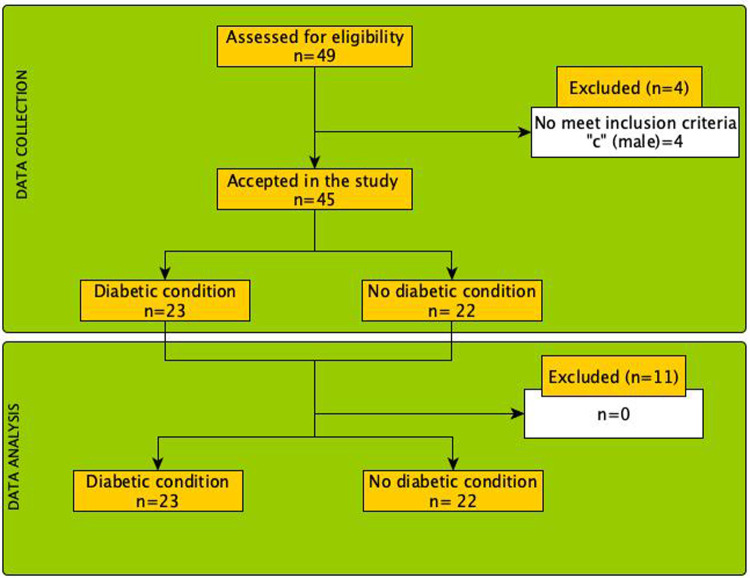
Flow diagram of the process of selection and analysis of the patients included in the present study.

Of the forty-nine (*n* = 49) participants with potential to be evaluated in this study, finally forty-five (*n* = 45) patients were recruited. Four participants were excluding due to no meet the inclusion criteria “c”. Therefore, a total of twenty-three participants (*n* = 23, 51%) were categorized as a diabetic condition, being twenty-two participants (*n* = 22, 49%) non-diabetics (see Figure 4).

Although non-diabetic patients took less time to complete bone consolidation (range between 24 and 40 days) after the intervention, the Mann-Withney U did not reveal statistically significant differences (*p* = 0.409, ES = 0.14 [-0.20 to 0.45]) in the comparison of consolidation time between patients with diabetes vs. non-diabetic patients. The mean and SD of consolidation time are summarized in Table 1.

On the other hand, a statistically significant relationship was found (*r* = 0.71, R^2 ^= 50%, *p* < 0.001) between the period of bone consolidation and days of discharge, as well as age and days of discharge (*r* = 0.36, R^2 ^= 13%, *p* < 0.001). However, no statistically significant relationships were found between pre-intervention glycemia scores and days to consolidation, nor discharge (*r* = 0.07, *p* = 0.646 and *r* = 0.07, *p* = 0.648, respectively).

**Figure 5:**
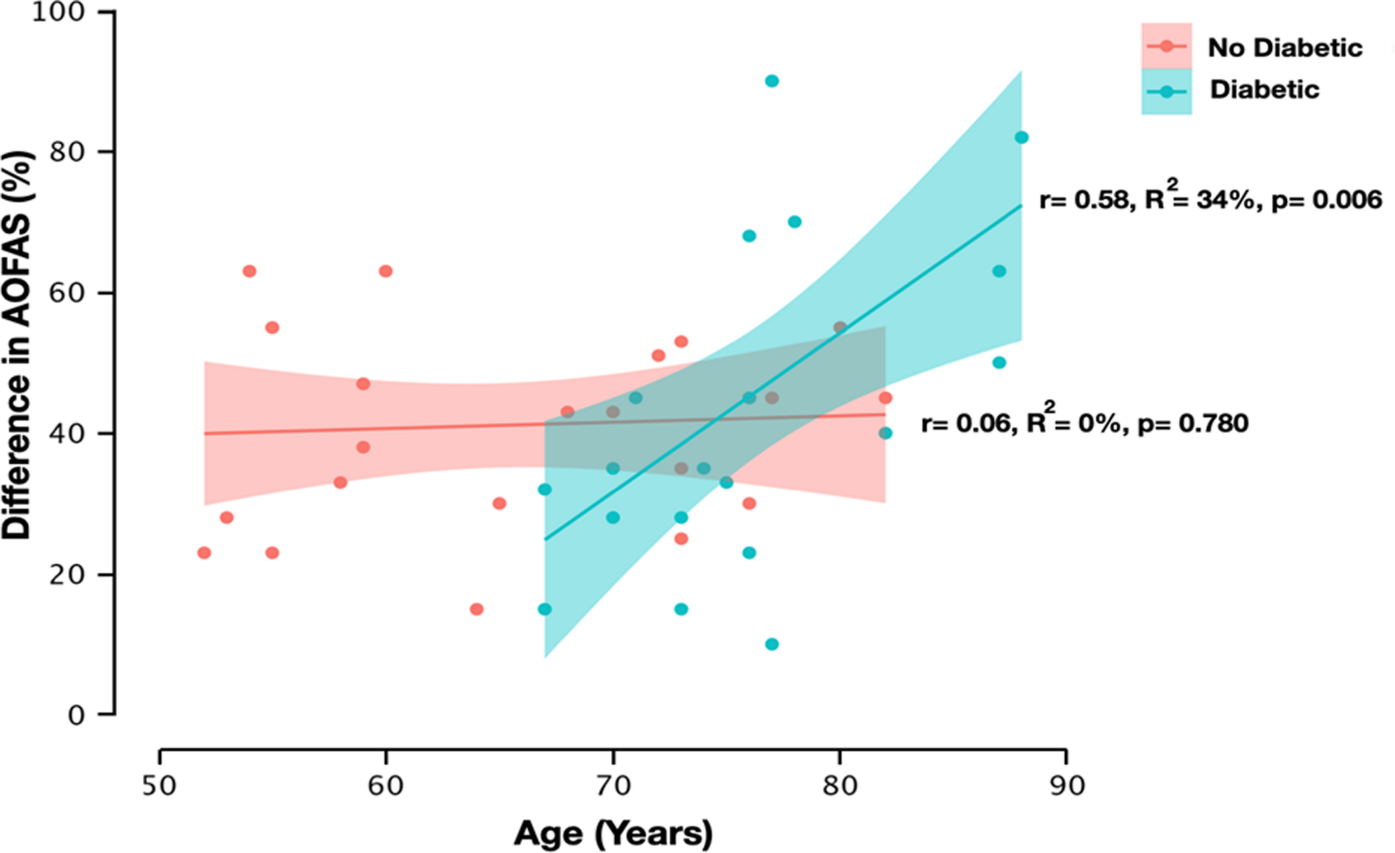
Relationship between American Orthopedic Foot and Ankle Surgery Society (AOFAS) test difference scores and age as a function of patients’ condition (i.e., diabetic vs. non-diabetic). The red dots reflect the AOFAS test difference scores in the non-diabetic condition, while the green dots reflect the AOFAS test difference scores in the diabetic condition. The shaded portion around the regression line reflects the 95% confidential interval. *r* = Pearson's correlation coefficient, *R*^2 ^= coefficient of determination, *p* = *p*-value.

Finally, ANCOVA of the percentage improvement in AOFAS test scores and diabetes status (i.e., diabetic vs. non-diabetic) showed statistically significant differences, both in the main effect of diabetes (F_[1,41] _= 9.41, *p* = 0.004) and in the diabetes × age interaction (F_[1,41] _= 9.17, *p* = 0.004). Figure 5 details the relationship between the percentage change in AOFAS scores and age.

## Discussion

The main objective of the present study was to describe and compare the bone consolidation time after osteotomy in diabetic vs. non-diabetic patients. The main finding of this study was that, regardless of the diabetic condition, the consolidation time was similar between both groups, showing no statistically significant differences and trivial effect size. The glycemia of the two groups was similar, being the group with diabetes a low-risk group, and for this reason we cannot determine the delay in consolidation as other studies have done (22).

On the other hand, a small but statistically significant relationship between patient age and discharge time was observed. Similarly, a statistically significant relationship was found between consolidation time and discharge days. Finally, it could be observed that as a function of diabetes status, the degree of functionality and age correlated in a statistically significant way in diabetic patients. The findings of this study provide evidence in favor of deformity correction the younger the patient and the better the metabolic control.

Nowadays, there are no specific classifications on the consolidation of osteotomies in intervened claw toes, what is described is the process of consolidation of bone fractures in general, based on scales already described for the formation of bone callus (36). In the absence of these classifications, Gerstenfeld et al. (24) carried out a review of histomorphometry methods in osteogenic processes in which they emphasized the need to study the bone repair process in detail to establish standardized criteria for evaluations that would serve to create a universal classification that would also detail observational tools for precise radiographic measurements (24).

Previous studies have shown that the evolution of fractures describes the existence of complications in the consolidation period related to smoking or age (37) although, loss of bone minerals or diabetes highly influence the process. The most recent evidence (18, 20, 22, 38) have pointed out that the bone tissue of diabetic patients presents loss of collagen (21), a high percentage of AGE advanced glycation products and a worse quality of trabecular bone which could compromise, in addition, its mechanical properties (20, 39). Our results showed that bone consolidation time was similar between diabetic and non-diabetic patients (see Table 1), achieving a solid and effective consolidation in both groups. Interestingly, these findings could suggest that correct glycemic control as well as good choice of surgical technique could favor the results of interventions in all types of patients, including those with type 2 DM.

Recently, foot minimally invasive surgery has gained popularity in DM patients (40, 41). The scarcity of scarring or tissue damage, the low number of complications (33, 42) as well as the absence of osteosynthesis reduce the number of infections while could favor the recovery time (13, 43). Several studies have pointed out that, given the existence of these techniques (i.e., minimally invasive), surgical interventions in diabetic patients should not be extended to the threat of loss of parts or tissues, since they could be used preventively (13, 44). Postponing interventions, especially in patients who have presented ulcers or suffer from reulcerations, could increase the risk of non-union of osteotomies. For example, a study leaded by Tamir et al. (43) showed that 6 months after surgery some patients failed to unite, although this lack of bone callus formation was asymptomatic. In 2003, Armstrong and Frykberg (45), published a study mainly aimed to classify diabetic foot surgery performed in the absence of critical limb ischemia considering three variables: the presence or absence of neuropathy, ulceration, and/or acute infection with risk of limb loss. The objective was to classify surgery based on the increasing order of amputation from I to IV. Our proposal in this study is related to class I or elective surgery, which includes those procedures performed to treat a painful deformity without loss of protective sensibility and class II, prophylactic surgery, formed by procedures performed to reduce the risk of ulceration (45).

The osteotomies performed for the correction of claw toes in patients operated in this study required weekly monitoring, not only for fluoroscopic assessment of the bony evolution, but also for the change of the post-surgical dressing. In this regard, percutaneous techniques require delicate management of the dressing. Previous studies have shown that the meticulousness of the dressing benefits the correct alignment of the fingers in the absence of osteosynthesis (33, 42). The compression of these bandages and their traction favor the formation of bone and fibrous tissue based on Roux's Law, which relates bone remodeling to the adaptive process of cells influenced by local stresses in the area. This application, which avoids osteosynthesis, also avoids infections that are sometimes triggered by metal fixations (13) and the final consolidation time does not vary between their use or not; while in the present case no infections have developed and total consolidation without osteosynthesis has been 4 to 6 weeks, other findings point to the removal of kirschner wires at 4 to 5 weeks (46).

There are several limitations to this study that should be keep in mind when interpret the findings. First, although no differences were found regarding bone consolidation time between diabetics and non-diabetic patients, we only included a sample of females' patients. Further studies including both sexes are necessary. In addition, the sample was carefully biased so that all patients in the study had tightly controlled glucose levels, including those of diabetic patients, so that the conditions between the two groups were as similar as possible for assessing the influence of balanced pathology.

## Conclusions

The bone healing time of claw toes operated by osteotomies using the minimal incision technique is almost similar between diabetic (controlled) and non-diabetic patients. In both type of patients, the length of discharge was statistically related to the days of consolidation: the faster the consolidation, the shorter the days of medical discharge. Finally, the degree of functionality after the operation correlated statistically significantly with age, only for DM patients.

## Data Availability

The raw data supporting the conclusions of this article will be made available by the authors, without undue reservation.
